# Enhanced Short-Term Memory Plasticity of WOx-Based Memristors by Inserting AlO_x_ Thin Layer

**DOI:** 10.3390/ma15249081

**Published:** 2022-12-19

**Authors:** Juyeong Pyo, Hoesung Ha, Sungjun Kim

**Affiliations:** Division of Electronics and Electrical Engineering, Dongguk University, Seoul 04620, Republic of Korea

**Keywords:** neuromorphic, resistive switching, tungsten oxide, short-term memory

## Abstract

ITO/WO_x_/TaN and ITO/WO_x_/AlO_x_/TaN memory cells were fabricated as a neuromorphic device that is compatible with CMOS. They are suitable for the information age, which requires a large amount of data as next-generation memory. The device with a thin AlO_x_ layer deposited by atomic layer deposition (ALD) has different electrical characteristics from the device without an AlO_x_ layer. The low current is achieved by inserting an ultra-thin AlO_x_ layer between the switching layer and the bottom electrode due to the tunneling barrier effect. Moreover, the short-term memory characteristics in bilayer devices are enhanced. The WO_x_/AlO_x_ device returns to the HRS without a separate reset process or energy consumption. The amount of gradual current reduction could be controlled by interval time. In addition, it is possible to maintain LRS for a longer time by forming it to implement long-term memory.

## 1. Introduction

The conventional digital computing system based on the von Neumann architecture has significantly developed in the past few decades, leading humans into the information age. Shortly, with the development of technologies such as artificial intelligence (AI) and the Internet of Things (IoT), the amount of data and information used in computing systems will exponentially increase [1,2]. However, the computing system based on the von Neumann structure is not suitable for handling large amounts of data in the Big Data era because of the structure that separates the computing part and the memory part in the computing system and the high-power consumption [3,4]. Thus, new memory devices should be developed to replace conventional memory to enhance the computing system.

A neuromorphic system imitates the human brain or a biological system [5,6,7,8]. Unlike von Neumann’s architecture, a neuromorphic computing system consumes low energy by parallel processing similar to the connections that link neurons and synapses in parallel [9,10,11]. It is necessary to understand and imitate biological synapses for the implementation of a neuromorphic system. The human brain has two types of synaptic plasticity to maintain memory: short-term plasticity (STP) and long-term plasticity (LTP). It is possible to emulate bio-synaptic simulation in the RRAM devices by the input pulse repetition, pulse amplitude, and interval time [12,13]. This is very similar to the biological system in which the pre-synapse releases the synaptic transmitters, travels across the pre-synaptic transmission, passes through the post-synapse, and finally enters the post-synapse.

The memristor is an electron memory device that can simulate artificial synapses [14,15,16]. Among several categories, resistance change-based new memory types, such as magnetic random-access memory (MRAM), ferroelectric random-access memory (FRAM), phase-change random-access memory (PRAM), and resistive random-access memory (RRAM), have been studied [17,18,19,20]. The mechanism of the MRAM device is based on the manipulation of the magnetization state through the spin-transfer torque effect. The FRAM causes the transition of a ferroelectric material by the voltage bias to lead to a change in the conductivity. The switching of the PRAM device is based on the change in the crystallinity of phase-change materials [21,22,23].

The RRAM device consists of a simple metal-insulator-metal (MIM) structure with a lot of materials including metal oxides and metal nitrides [24]. Among them, metal oxides such as HfO_x_, TaO_x_, and WO_x_ are promising due to their superior memory performances. It maintains a low resistance state (LRS) in a specific voltage range and a high resistance state (HRS) in a different voltage range. The process of switching from HRS to LRS in the RRAM cell by applying the turn-on voltage is called “set”, and the process of switching from LRS to HRS by applying the turn-off voltage is called “reset” [25]. The set process is based on the phenomenon in which conducting filaments (CF) form inside the insulator or switching layer between the top electrode (TE) and bottom electrode (BE). In contrast, the reset process is based on a phenomenon in which the CF disappears. When applying extremely high voltages on the RRAM device, excessive CF can be formed, and the device no longer serves as a memory. Compliance current (CC) should be applied during the set process. If the CC is set up before performing a set switching, the current does not increase above a threshold level. It does not prevent a permanent breakdown of the device but also allows the CF to be resized. A larger CC produces larger filaments during the switching, and hence larger voltages are required to destroy and reconfigure the filaments.

Short-term memory (STM) is a temporary potentiation/depression of neural connections. Unlike long-term memory, which lasts in the range of hours to years, an STM typically lasts for seconds to tens of minutes. Owing to the volatility of the memristors, short-term plasticity can be implemented in various memristors [26]. In addition, STP can be converted to LTP through iterative experience, which is related to stimulus in a synaptic structure.

In this study, we fabricated the synaptic behaviors of WO_x_/AlO_x_-based RRAM devices for neuromorphic systems. ITO as TE and TaN as BE are compatible with the complementary metal-oxide-semiconductor (CMOS) fabrication process. The combination of a conductive oxide metal (ITO) and a metal (TaN) can control the WO_x_ and AlO_x_ layer for resistive switching by oxygen exchange. The control device was prepared without AlO_x_, and the device with a ~2 nm thickness between the WO_x_ layer and TaN layer to make a difference in electrical characteristics by the introduction of an ultra-thin high-k film. Previous studies have reported the introduction of an ultra-thin high-k film, for example, HfO_2_ and AlO_x_ films [27,28,29]. However, there are limited studies on the bi-layer of AlO_x_ and metal-oxide for STM. This study reports the effects of an AlO_x_ layer on STM and its advantages as a neuromorphic device.

## 2. Materials and Methods

We fabricated both device 1 (D1) and device 2 (D2) on SiO_2_/Si substrates. The structure of D1 was ITO/WO_x_/TaN, and the structure of D2 was ITO/WO_x_/AlO_x_/TaN. D1 and D2 have similar fabrication processes except for the AlO_x_ layer in D2. First, in all the processes, ~100 nm of TaN, BE, was deposited by a company (GMEK, Anyang, Republic of Korea). The Ta target was reactively sputtered with Ar (19 sccm) and N_2_ (1 sccm) at room temperature (RT). After that, only in the case of D2, ~2 nm of the AlO_x_ thin film was grown before the WO_x_ layer deposition through an atomic layer deposition (ALD, ISRC, Seoul, Republic of Korea) system. The trimethylaluminum (TMA) precursor was reacted with H_2_O at 150 °C. Next, a ~60 nm thickness of the WO_x_ switching layer (by GMEK) was formed at RT and 1 mTorr condition using reactive sputtering with Ar (8 sccm) and O_2_ (12 sccm) with W target. Finally, the ITO electrode was patterned using an e-beam evaporator (KANC, Suwon, Republic of Korea). The pattern used a shadow mask, and the cell size was 100 × 100 μm^2^ dot.

To confirm the composition of the ITO/WO_x_/AlO_x_/TaN device, advanced scanning transmission electron microscopy (STEM; Hanyang Univ., Seoul, Republic of Korea) was performed on the cross-section from TE to BE of the D2 sample with a focused ion beam (FIB). The electrical resistive switching and pulse measurements to mimic the synapse were measured using the Keithley 4200 SCS semiconductor parameter analyzer (Tektronix Inc., Beaverton, OR, USA) and the 4225-PMU ultrafast current voltage pulse module, respectively. While applying voltage bias to ITO TE, the ground was connected to TaN BE. In direct current (DC) mode, the voltage gradually increases by 0.05 V for each step to set/reset voltage, whereas, in pulse mode, it instantaneously increases from 0 V to a predefined amplitude without a step.

## 3. Results and Discussion

**Figure 1:**
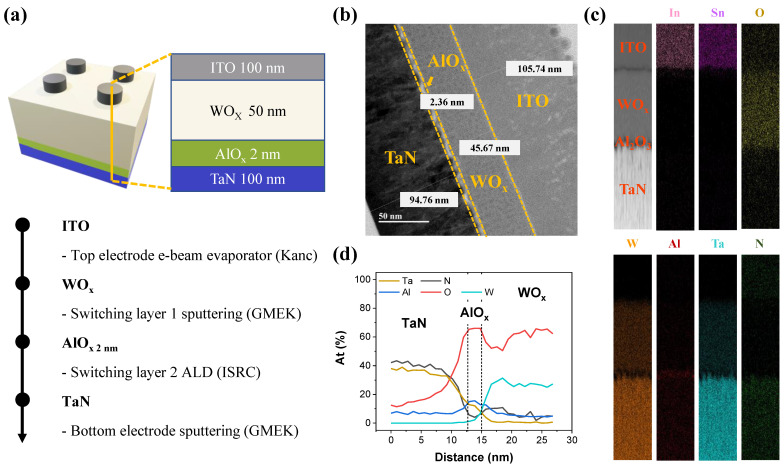
(**a**) The schematic showing the structure of ITO/WO_x_/AlO_x_/TaN device and the brief fabrication process; (**b**) STEM image; (**c**) EDS mapping image; and (**d**) Line scanning for the atomic ratio of the device.

Figure 1a shows the device stacking and fabrication process. The image shows the device prepared by sample cutting using FIB in the vertical direction from ITO to TaN. The TEM image shows a cross-sectional image of D2. The difference between the target thickness (~100 nm thick ITO, ~50 nm WO_x_, ~2 nm AlO_x_, and ~100 nm TaN) and deposited thickness is approximately ±5 nm when compared with the TEM image for each layer. Energy dispersive X-ray spectroscopy (EDS) was performed to analyze the components of D2. Figure 1c shows the EDS mapping for each element with different colors. For confirmation, as shown in Figure 1d, the atomic line scan was focused on the AlO_x_ area located between WO_x_ and TaN layers. At ~12 nm on the *x*-axis, the Ta (tan line) and the N (black line) indicate approximately 34% and 38% in the BE layer, respectively. Metal-TaN approximately contains a 1:1 ratio of Ta:N. Therefore, a metallic TaN layer was deposited [30]. The O ratio (red line) increases in the positive *x*-direction going from BE to the AlO_x_ layer; this is because the TaN layer is easily oxidized [31]. Near 12–15 nm on the *x*-axis, a ~2 nm AlO_x_ layer is observed with a much higher O ratio than the Al ratio [32]. The WO_x_ layer, another switching layer from 15 nm on the *x*-axis, is presented as the W (turquoise line) and the O (red line). The atomic ratio of W and O is approximately 25% and 60%, respectively. In the reported RRAM paper, the WO_x_ layer deposited by tungsten-reactive sputtering also contains 20–30% tungsten and 60–70% oxygen [33]. Overall, there is more oxygen than tungsten in the WO_x_ layer.

**Figure 2:**
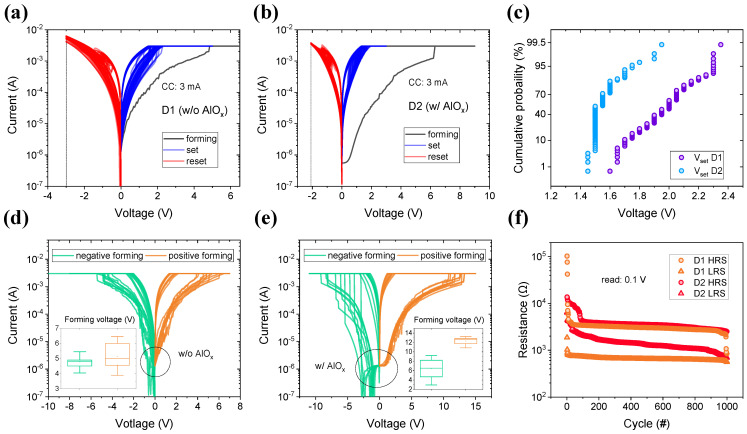
The I-V curve of (**a**) ITO/WO_x_/TaN device 1 and (**b**) ITO/WO_x_/AlO_x_/TaN device 2 during 100 cycles. The black lines indicate the forming process, the red lines indicate the reset process, and the blue lines indicate the set process. (**c**) The cumulative probability of set voltage. A blue circle group is device 1 and a purple circle group is device 2, respectively. The forming process of (**d**) device 1 and (**e**) device 2 was performed by negative or positive bias. Each inserted figure shows a forming voltage range at 3 mA. (**f**) Switching endurance of D1 (orange) and D2 (red) during 1000 cycles.

The electrical characteristics, including the resistive switching of the RRAM device, were analyzed by I-V curves, as shown in Figure 2a,b. D1 (w/o AlO_x_) and D2 (w/o AlO_x_) were prepared to investigate the effect of 2-nm thin AlO_x_ film on resistive switching and synaptic characteristics. The tunneling effect can occur since the AlO_x_ film is ultra-thin; however, when a relatively high voltage such as the forming voltage is applied, the device with the AlO_x_ layer undergoes a soft breakdown, and the tunneling effect is no longer considered. The tunneling effect will be discussed in detail later. The switching after filament formation inside the switching layers is shown in Figure 2. A forming voltage (9 V) was applied to TE with a CC of 3 mA, which was used to prevent breakdown, inducing filament formation. Both D1 and D2 show similar switching curves after the forming process. This indicates that the switching characteristic of the WO_x_ layer is more dominant after the filament is generated inside the very thin AlO_x_ layer during the conducting filament formation process. Although D1 and D2 have the same thickness for the WO_x_ layer, some differences such as set and reset voltage and forming I-V curve are observed, owing to the ultra-thin AlO_x_ layer.

The reset voltage, which is the maximum voltage, allows the switching operation. When −2.1 V is applied to D2, a stable reset process occurs within 100 cycles, whereas D1 requires a higher voltage of −3 V. The set voltage, where the current becomes 3 mA which is the same CC with the forming process, was plotted in Figure 2c. D1 (1.6 to 2.35 V) has a relatively wider distribution than D2 (1.45 to 1.95 V) during the 100 cycles. This indicates that D2 has a narrower distribution and better uniformity than D1. Figure 2f shows the endurance of HRS and LRS at the read voltage of 0.1 V for 1000 cycles. D1 maintains the LRS and HRS for 1000 cycles, except for changes in the early and later switching. D2 also maintains HRS and LRS, but the window that is smaller than D1 gradually grows during switching.

I-V curves confirmed that the difference in resistive switching depends on the ultra-thin AlO_x_ layer after the forming process. The thin AlO_x_ layer can affect the filament in the thick WO_x_ layer. The forming curves are limited by CC of 3 mA for the cell-to-cell of D1 and D2 in Figure 2c,d, respectively. In the voltage sweep region (from −1 V to 1 V) in these two curves, the current of D1 increases with the voltage without significant suppression, whereas the current of D2 does not increase in a particular voltage region. The energy band gap (E_g_) of AlO_x_ and WO_x_ are 8–8.8 eV and 3.0–3.2 eV, respectively [34,35,36]. Many oxygen vacancies exist inside the WO_x_ layer because WO_x_ was deposited by physical vapor deposition (PVD). The AlO_x_ grown by ALD, which is a layer-by-layer process, has small defects such as oxygen vacancies inside the grown layer. Therefore, to form a filament inside AlO_x_, a higher voltage than WO_x_ is commonly required, and it appears as the current-suppressed area in the forming curve. It can also be identified by the inserted forming voltage. The negative forming voltage of D1 (D2) is −5.45 to −4.05 V (−9.2 to −3.95 V) and the positive forming voltage is 3.9 to 6.45 V (10.85 to 13.25 V). The minimum voltage of D1 is smaller than that of D2 for soft breakdown to work when switching is possible as RRAM.

**Figure 3:**
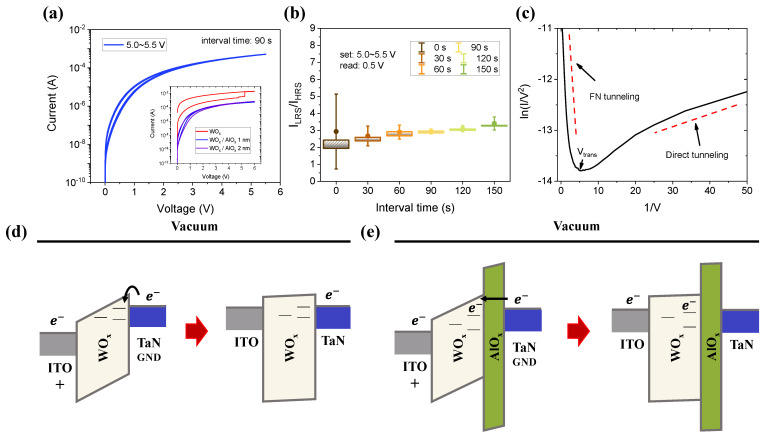
(**a**) The I-V curve for set switching before forming process. Inserted figure shows how each device (AlO_x_: 0, 1, 2 nm) operates in a small positive bias. (**b**) Before forming device 2, plotting the current ratio of LRS/HRS with interval time. The interval time is the delay time from set to set. Device 2 works by tunneling effect since the ultra-thin AlO_x_ layer is high-k dielectric. (**c**) The Fowler–Nordheim tunneling and direct tunneling plot of device 2. (**d**) The energy band model of device 1 and (**e**) device 2 for set switching.

Even before the forming process, switching characteristics significantly vary based on the AlO_x_ layer. When a small voltage (<3.9 V) is applied to D1 without AlO_x_, it does not change from HRS to LRS. Above 3.9 V, the soft breakdown in the WO_x_ layer may or may not occur. On the contrary, when ~6 V is applied to D2 with AlO_x_, as shown in the insert figure of Figure 3a, the set switching from HRS to LRS occurs. Compared with D1, which undergoes non-switching at a small voltage, D2 is distinguishable between HRS and LRS. In addition, the short-term effect of returning to HRS is apparent. To analyze short-term memory characteristics, D2 was set to an interval time of 90 s between 5.0 and 5.5 V applied voltages, which is enough time to return to the HRS. The HRS/LRS ratio is well maintained regardless of the voltage amplitude, and the shift from HRS to LRS is not significant. The degree of shift is shown in Figure 3b. The processing steps including voltage are the same as those in Figure 3a, but the interval time is set differently to 0, 30, 60, 90, 120, and 150 s. As a result, the I_LRS_/I_HRS_ fluctuates the most by 2.03 to 7.2 at 0 s and fluctuates the least by 2.85 to 3.21 at 90 s.

The short-term memory effects can be observed in the LRS in a small voltage regime for D2, unlike D1. This behavior probably occurs due to the ~2 nm AlO_x_ layer, which acts as a tunnel barrier. In D2 with an ultra-thin layer, the switching mechanism can be explained as a tunneling effect: Fowler–Nordheim tunneling (FNT) or direct tunneling (DT) [37,38,39]. The relationship equation between tunneling current (I) and voltage (V) is as follows:

(1) $$I\propto Vexp\left(-\frac{2d\sqrt{2m^{*}\Phi_{B}}}{h}\right):\;DT\;(V<V_{trans})$$

(2) $$I\propto V^{2}exp\left(-\frac{4d\sqrt{2m^{*}\Phi_{B}^{3}}}{3heV}\right):\;FNT(V>V_{trans}),$$

where d is the insulator thickness; $m^{*}$ is the effective mass of an electron; $\Phi_{B}$ is the barrier height; h is Planck’s constant; and $V_{trans}$ is the transition voltage from DT to FNT. DT is dominant at a very small voltage (<$V_{trans}$). DT is not affected by the bias voltage but is influenced by the thickness of the switching layer (~2 nm AlO_x_), as indicated by Equation (1). FNT appearing at a relatively high voltage (>$V_{trans}$) is more influenced by voltage than thickness, as indicated by Equation (2). Therefore, in Figure 3c, the dominant current flows by a tunneling effect. D2 can convert the memory state with a small voltage even though there is no forming process inside the switching layer. In Figure 3d, when a small positive bias is applied to TE in D1, the electrons reach the BE by the formation of defects such as oxygen vacancies inside the WO_x_ layer; however, the oxygen vacancies do not affect the conducting filament in the device. D2 has an AlO_x_ layer located between the WO_x_ and TaN layers; thus, when a small bias positive is applied, the current flow is improved as a result of the oxygen vacancies in the WO_x_ moving toward AlO_x_. However, when the bias is removed, oxygen vacancies at the interface of WO_x_ and AlO_x_ are randomly dispersed returning eventually to the HRS [40].

**Figure 4:**
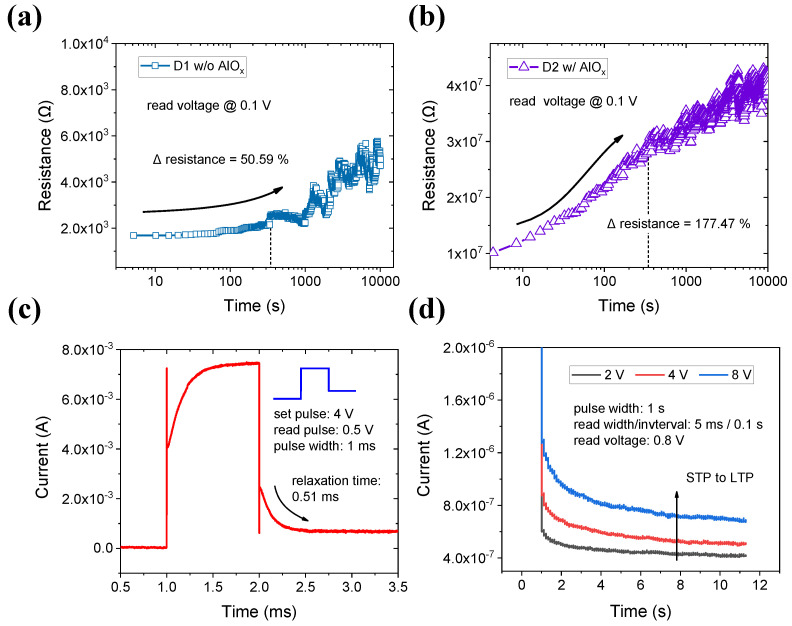
The retention of (**a**) device 1 after forming/reset/set switching and (**b**) device 2 after only set switching. Device 1 tries to maintain LTS, while device 2 has a short-term characteristic. In pulse mode, (**c**) the relaxation time and (**d**) STP to LTP control by pulse amplitude of device 2. The relaxation time is longer as the amplitude increases.

Additionally, retention and pulse measurements showing the short-term characteristics of devices were performed. The retention data of D1 show how long WO_x_ can maintain the LRS after the forming/reset/set process (Figure 4a). D2 identified whether the LRS can be maintained for a long time after the set process without forming the process in Figure 4b. As a result of reading at 0.1 V for 10,000 s in the LRS immediately after the set process, the resistance of D1 increased by 50% from the initial low resistance after 345 s: from 1.683 kΩ to 2.535 kΩ. At this point, the resistance of D2 increased by 177.47% from 10.1 MΩ in the initial LRS to 28.1 MΩ. The fact that the LRS of two devices rises indicates returning to the HRS over time. Although forming bias and reset/set bias are applied to D1, it does not have good retention that preserves LRS or the “1” memory state. The weak retention is largely due to recombination between the oxygen and oxygen vacancies. The resistance of D2 significantly increases compared with that of D1 during the same time. The participating oxygen vacancies are less because the current caused by tunneling is due to the ultra-thin dielectric as well as the WO_x_ internal defects [41]. Therefore, by adding the AlOx layer, the operating current of the device can be lowered to improve the short-term memory characteristics.

To study the plasticity for performing the synapse function, the relaxation time was measured by following a read pulse after one pulse that increases the current of the device. In Figure 4c, after the current of the device is increased by one pulse with an amplitude of 4 V and a pulse width of 1 ms, the total time for the current by a read pulse of 0.5 V to return gradually to the HRS is approximately 0.51 ms. As shown in the DC retention result, even though only the read is applied without a pulse that increases the current, the current by the pulse is also recovered spontaneously. Synaptic plasticity is a fundamental concept in biological systems. The changes in the weight of synapses can be emulated by the increase or decrease in the conductance in RRAM, for example, STP/LTP and potentiation/depression. First, the ratio at which the current recovers to its initial state was measured with the pulse amplitude. The larger the amplitude of the pulse, the lower the recovery speed. As illustrated in Figure 4d, at 2 V, the current does not increase significantly, and hence, the relaxation time is also short. On the contrary, as the voltage amplitude increases to 4 V or 8 V, the current visibly increases, which results in a longer relaxation time. In the end, the stronger the stimulus, the greater the LTP than the STP, which is similar to a strong impression in the human brain or biological systems. However, when a significantly large amplitude is applied to the device, D2 eventually becomes forming and works in the same state as D1. Applying several pulses to D2, which is a short-term device with no switching failure to imitate synaptic potentiation and depression, suggests its suitability as a neuromorphic device.

**Figure 5:**
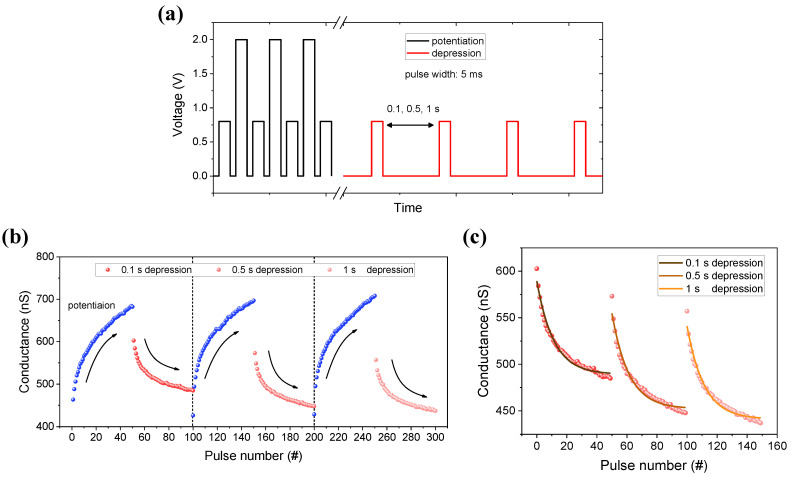
(**a**) An input pulse schematic for potentiation and depression. The potentiation pulses (black) are designed so that the pulse width is 5 ms and amplitude 2 V. The depression pulses (red) show the read pulse of 0.8 V that the interval time is only different by 0.1, 0.5, 1 s. (**b**) The conductance change of potentiation (blue)/depression (red). (**c**) A graph of the 0.1, 0.5, 1 s depression area fitting by the equation, respectively.

Figure 5b shows a time-dependent pulse schematic of potentiation/depression. The black line represents the synaptic potentiation, and the red line represents the synaptic depression with an amplitude of 2 V, a width of 5 ms, and a read of 0.8 V. The conductance increases with a pair of read and positive pulses, but it decreases to only the read pulse without a negative pulse, using short-term characteristics. The depression is controlled by adjusting the interval time from reading to 0.1, 0.5, and 1 s. As a result, as shown in Figure 5a, the conductance that performs the synaptic weight sufficiently potentiates and depresses differently depending on the interval time. When the degree of depression is fitted with an exponential decay equation, as shown in Figure 5c, the factors that determine the slope are $1.00\times 10^{-7}$ (0.1 s), $8.59\times 10^{-6}$ (0.5 s), and $6.54\times 10^{-4}$ (1 s), respectively. Therefore, the reduction in conductance also increases as the time between the reads increases.

**Figure 6:**
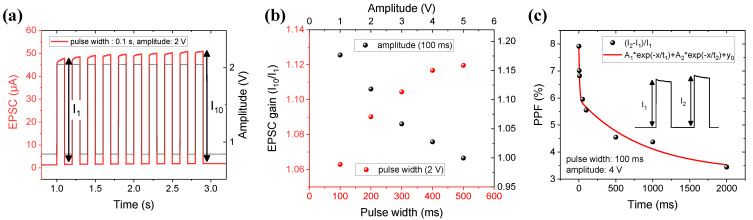
(**a**) EPSC fired by repeated pulses with short interval time that is before returning to the initial state. I_1_ and I_10_ are the currents triggered by the first pulse and the tenth pulse, respectively. (**b**) EPSC gain (I_1_/I_10_) plotted according to pulse width and pulse amplitude. Fixed 2 V at different pulse widths (from 100 ms to 500 ms), on the other sides, fixed 100 ms at different pulse amplitude (from 1 V to 5 V). (**c**) PPF (%), as a definition of (I_2_−I_1_)/I_1_, illustrated current increasing due to the interval time of a paired-pulse.

Signals are transmitted by a neurotransmitter between pre-synapse and post-synapse. As it is transmitted, the weight of the synapse changes as it is activated. The TE performs presynaptic works and the BE performs postsynaptic works controlling the conductivity (weight) of the device. The conductivity change of the device is defined as an excitatory postsynaptic current (EPSC). As shown in Figure 6a, the EPSC is implemented by applying a pulse width of 0.1 s and an amplitude of 2 V to TE. The black line indicates the pulse schematic, and the red time indicates the reacted EPSC. The conductive filament in bulk generated by the first pulse is then enhanced by the next pulse. As 10 pulses are input, in Figure 6b, the EPSC is the same as accumulated. Comparing the first pulse with the 10th pulse, the EPSC gain (I_10_/I_1_) improves from 1.06 to 1.12 when the pulse width (fixed 2 V) lengthens from 100 ms to 500 ms. On the other hand, as the amplitude (fixed 100 ms) enlarges from 1 V to 5 V, the EPSC gain decreases from 1.18 to 1.00. In a large amplitude, the conductivity significantly accelerates by the first pulse, so the 10th conductivity is insignificant.

The EPSC of D2 with the short-term characteristic slowly decays to a steady state when the stimulus is removed. Therefore, the change in that magnitude is different by the interval time of the pulses. This effect is paired-pulse facilitation (PPF). The PPF is defined by Equation (3) which indicates how the current is triggered by the paired pulse.

(3) $$PPF\;\left(\%\right)=100*\left(I_{2}-I_{1}\right)/I_{1}$$

Here, *I*_1_ denotes a current increased by the first stimulus, and *I*_2_ denotes a current increased by the second stimulus. Figure 6c show the PPF index by varying the interval time from 1 to 2000 ms for D2. During a very short interval time, the second current increased relatively, but as the interval time is longer, the increase gradually decreased. As a result, the decaying conductivity is controlled by adjusting the interval time due to the characteristics of recovering to the steady state of D2.

## 4. Conclusions

Herein, we studied the ITO/WO_x_/AlO_x_/TaN device in which an ultra-thin film of AlO_x_ was inserted between WO_x_ and TaN in the ITO/WO_x_/TaN device. First, STEM and EDS mapping analyses were used to confirm the components of the device. Comparing the I-V and forming curves for the two devices, the WO_x_/AlO_x_-based device had lower energy consumption and higher endurance. The current is suppressed due to AlO_x_ with a large band gap, so low-current switching is achieved by the tunneling barrier. This low current improves the short-term characteristics of the WO_x_-based device. In the pulse mode, the increased current returns to a high resistance state over time, and its relaxation time varies depending on the amplitude. This relaxation time also can be controlled by adjusting the interval between the read pulses. Thus, the implementation of flexible potentiation and depression is possible without an additional reset process using AlO_x_.

## Data Availability

Not applicable.
